# Fetal Hydrops: Genetic Dissection of an Unspecific Sonographic Finding—A Comprehensive Review

**DOI:** 10.3390/diagnostics15040465

**Published:** 2025-02-14

**Authors:** Gioia Mastromoro, Daniele Guadagnolo, Alessandro De Luca, Mauro Ciro Antonio Rongioletti, Antonio Pizzuti

**Affiliations:** 1 Department of Laboratory Science, Ospedale Isola Tiberina—Gemelli Isola, 00186 Rome, Italy; maurociroantonio.rongioletti@fbf-isola.it; 2 Department of Experimental Medicine, Sapienza University of Rome, 00185 Rome, Italy; daniele.guadagnolo@uniroma1.it (D.G.); antonio.pizzuti@uniroma1.it (A.P.); 3 Medical Genetics Division, Fondazione IRCCS Casa Sollievo della Sofferenza, 71013 San Giovanni Rotondo, Italy; a.deluca@operapadrepio.it

**Keywords:** fetal hydrops, prenatal diagnosis, molecular genetics, prenatal exome sequencing

## Abstract

Fetal hydrops is defined as the presence of abnormal fluid collections in two or more intra-fetal compartments. It has been classified based on etiology (immune vs. non-immune), on the presence or absence of other findings (isolated vs. non-isolated) and on the gestational age at presentation (first-, second- or third-trimester). In all cases of non-immune hydrops fetalis, invasive prenatal diagnosis is offered. However, after cytogenetic analyses, 80% of fetuses remain without etiological diagnosis, not allowing one to define the prognosis and to formulate recurrence risks. Several geneticists recommend performing either a next-generation sequencing panel (commonly limited to RASopathy testing) or exome sequencing, if cytogenetic tests are inconclusive. In the literature, the data are extremely heterogeneous, due to the differences in these indications and the limitation of study to a select group of genes. The identification of the underlying cause is crucial, as prognostic information and even therapy options are becoming increasingly available for a wide and growing array of genetic conditions. A systematic approach would allow an overall evaluation of the diagnostic rate of the exome sequencing in fetal effusions, also calculating the prevalence of associated diseases, with the aim of obtaining a diagnosis, defining the most appropriate management for each case, and broadening the spectrum of conditions known to be associated with hydrops.

## 1. Introduction

Fetal hydrops is detected through prenatal sonography in 1:1700 pregnancies [1] and it is defined as the presence of abnormal fluid collections in two or more intra-fetal compartments [2]. The involved body areas are represented by the serosa and the subcutaneous tissue [2]. This condition is associated with high risk of fetal demise and perinatal complications or death [2,3,4,5]. Quantitative alterations of amniotic fluid are not a primary feature of fetal hydrops, but can add to the clinical picture as secondary [2].

Fetal hydrops is not a single nosological entity, but rather the possible result of different conditions causing fetal fluid imbalance [6]. Historically, hydrops fetalis has been classified based on the presence or absence of other findings (isolated vs. non-isolated), on the gestational age at presentation (first-, second- or third-trimester) and on the underlying etiology. In particular, a distinction is made between immune hydrops fetalis and non-immune hydrops fetalis (NIHF), with and without maternal antibodies directed against fetal erythrocyte antigens, respectively.

Fetal hydrops is usually regarded as a severe finding, with fetal, neonatal and maternal risks. There is an increased risk of miscarriage, intrauterine fetal demise and stillbirth [2]. An accurate quantification of the risks is difficult due to the differences in enrollment criteria for literature cohorts and to the legal and social variabilities in the access to pregnancy termination. Fetal risks are determined by the underlying cause, by the possibly associated anomalies, by the secondary hemodynamic imbalance and by the possible obstetric complications [2,6,7]. Secondary involvement of extra-fetal fluids (e.g., polyhydramnios) can result in preterm birth, which occurs in 2/3 of cases [2,6,7]. After birth, depending on the hemodynamic balance and underlying cause, some cases can show a regression of the phenotype, while others are progressive and present poor prognosis. Neonatal complications can include pleural/pericardial effusions, ascites, heart failure and pulmonary hypoplasia. A specific maternal pregnancy-related systemic condition, known as mirror syndrome or Ballantyne’s syndrome, can be detected in a variable fraction (5–29%) of cases [3,8]. It is characterized by variable maternal systemic and placental edema, with possible cardiovascular involvement and obstetric risks. The pathophysiology shares some clinical features and molecular pathways with preeclampsia, showing a dysregulation of placenta-mediated angiogenic and antiangiogenic factors [9], but it differs for the presence of maternal hemodilution [10]. Cases have been reported in the literature in which, after reversing the cause of fetal hydrops, there was an improvement in the maternal clinical conditions. As of today, no universal or specific treatment is available for fetal hydrops, but supportive care can reduce some of the complications. Antenatal steroid administration to reduce the risks of preterm birth is commonly performed. Antiarrhythmic drugs can be administered to the mother in case of fetal arrhythmias. Fetal transfusions can be performed in anemia-related cases. In utero fetal effusion diversion (thoracentesis, pericardiocentesis, paracentesis) can be performed to reduce the associated morbidities.

To date, in many cases, the underlying cause of fetal hydrops is not found. This entails additional risks for both the pregnant woman and the fetus, as it does not allow the most suitable management to be organized for the specific case, nor to estimate recurrence risks for the couple.

## 2. Developmental Pathology

Intra-fetal fluid collections can manifest when the homeostatic mechanism that regulates the production of capillary filtrate and its reabsorption through the lymphatic system fails. This makes hydrops the consequence of a dynamic process of pathological fluid accumulation [2,11]. The factors involved in interstitial fluid homeostasis are capillary permeability, capillary hydrostatic pressure, plasmatic oncotic pressure, lymphatic vessel fluid reabsorption and interstitial compliance [2,6,7]. Which of these is the predominant mechanism usually depends on the etiology of the hydrops; however, the other components of the system can be secondarily impaired as a result of the accumulation of fluids.

In fetal hydrops, the dysregulation of capillary filtration and fluid reabsorption occurs on a systemic level. The characteristics of prenatal microcirculation and the lymphatic system make fetuses more prone to develop interstitial fluid accumulation, due to a higher capillary permeability, a more compliant interstitial compartment and the greater influence of venous pressures on lymphatic return. 

Hydrops fetalis occurring in the first trimester is not related to the presence of maternal antibodies, as a consistent transplacental passage of erythrocytes does not happen at this stage. This early sonographic finding is a negative prognostic factor, leading to a high risk of miscarriage.

A more prevalent finding, possibly associated with fetal hydrops, is represented by septated first-trimester cystic hygroma. It is a lymphatic developmental anomaly detected in 1:285 fetuses [12], where the high-protein fluid is usually located in the nuchal region, and can extend along the entire length of the fetus and/or laterally [13].

Placenta involvement, resulting in edema or increased amniotic fluid, can be part of the sonographic picture, but without these being parameters for diagnosis.

### 2.1. Immune Hydrops Fetalis or Erythroblastosis Fetalis

Immune hydrops fetalis occurs when maternal anti-erythrocyte antibodies result in high fetal red blood cell clearance, with a compensatory, but insufficient, increase in extramedullary hematopoiesis (with release of erythroblastic precursors in the blood flow) and subsequent fetal anemia [2]. Severe anemia (hemoglobin, Hb, <5 g/dL) is associated with the multifactorial development of hydrops, due to high heart output, hypoxia, which triggers intra-fetal fluid retention, subsequent congestive heart failure and an increase in capillary hydrostatic pressure [2,6]. The most common form of immune fetal hydrops is due to Rh-D alloimmunization. More rarely, anti-c, C, e, E, Duffy antibodies, Kell and ABO alloimmunization are reported [14]. Sonographically, it is possible to evaluate the presence of fetal anemia with high-sensitivity measuring of the fetal middle cerebral artery peak systolic velocity (MCA-PSV) [15,16,17].

Historically, a large number of hydrops fetalis cases have been recognized on an immunological basis, but since the introduction of Rh immunoglobulin prophylaxis, immune fetal hydrops seem to represent 10–20% of the total [18].

### 2.2. Non-Immune Hydrops Fetalis

The incidence of NIHF ranges from 1:1500 to 1:3800, accounting for about 80–90% of HF [18], and it is defined by the presence of two or more fluid collections without circulating antibodies against erythrocytes in the maternal serum.

NIHF is a heterogeneous condition, whose etiology is extremely variable: we can distinguish genetically determined causes and non-genetically determined causes. The first group includes chromosomopathies (Monosomy X, Trisomy 21, Trisomy 13, Trisomy 18), inborn errors of metabolism and storage disorders (for example, Niemann–Pick disease type C, Gaucher disease type 2, infantile sialic acid storage disease, mucopolysaccharidosis type VII, glycogen storage disease IV) [19], alpha thalassemia, congenital anemias (erythropoietic porphyria), lymphatic dysplasias, anomalies of erythrocyte membranes and other syndromic conditions. Non-genetically determined causes of NIHF are represented by cardiovascular malformations, maternal diseases (diabetes mellitus, hyperthyroidism), infections (cytomegalovirus, parvovirus B19, syphilis) [20] and by other causes.

**Figure 1:**
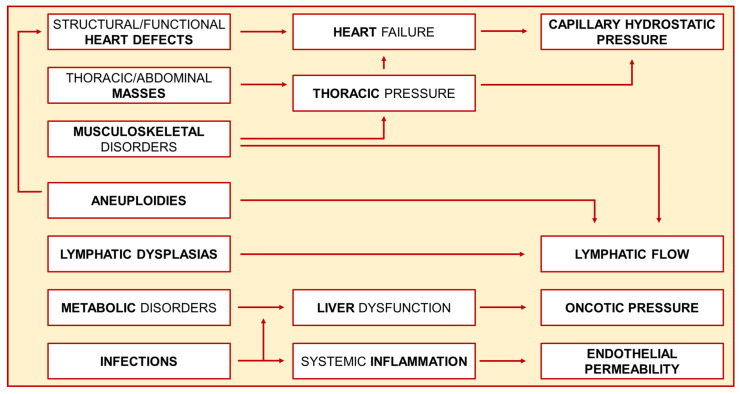
The pathophysiology of Non-Immune Hydrops Fetalis (NIHF). The figure shows how different causes converge towards common mechanisms in determining NIHF.

The underlying pathogenic mechanisms for NIHF can vary based on the cause, but ultimately converge towards key common factors (Figure 1).

For genetic conditions, the pathophysiology can be attributed to heart defects, to lymphatic developmental anomalies in chromosomal anomalies and monogenic lymphatic dysplasias, to abnormal oncotic pressure due to liver dysfunction in metabolic and lysosomal storage disorders, to anemia in thalassemia and erythrocyte anomalies, and to underlying cardiovascular malformations in conditions with structural defects. In cardiovascular anomalies (malformation and/or secondary fetal heart failure), the increased central venous and capillary pressure is regarded as the cause of hydrops.

For infectious disease, increased capillary permeability (due to endothelial damage) and anemia are considered the main causes for the fluid filtration/reabsorption imbalance [6,7].

In case of malformations of the left cardiac chambers, valve or aortic arch obstruction may occur, with a consequent increase in left intraventricular and intra-atrial pressure and a reduced or absent right/left shunt at the level of the foramen ovale. This results in an increase in right atrial pressure with systemic venous congestion and hydrops [21].

Non-structural cardiac causes that can induce hydrops are represented by arrhythmic episodes, especially tachyarrhythmias. The latter can be determined by lesions such as rhabdomyomas or by conditions due to the presence of ventricular pre-excitation due to atrioventricular accessory pathways (e.g., Wolff–Parkinson–White). Bradycardia may result from an abnormal cardiac morphogenesis or may be related to the presence of fetal atrioventricular block due to maternal anti-Ro/anti-SSA antibodies and anti-La/anti-SSB antibodies [2,6,7].

Sometimes, in genetically determined conditions, the cystic hygroma can represent the first ultrasound anomaly, which then extends and involves further body areas, developing fetal hydrops [22].

Other mechanical causes of fetal hydrops are represented by consequences of altered circulation in monochorionic twin pregnancies, including complications such as twin-to-twin transfusion syndrome and twin reversed arterial perfusion, and a compression effect caused by an intrathoracic or intra-abdominal fetal mass [6,7].

The anamnestic record of administration of indomethacin, other FANS or paracetamol during the third trimester of pregnancy can determine a transient vasoconstriction of ductus arteriosus, causing an increased central venous pressure and hydrops [23].

The stratification of the risk for chromosomal anomalies can be based on maternal age, family history and ultrasound parameters. Structural anomalies are strongly associated with genetic, and especially chromosomal, disorders [6,7,11]. Some ultrasound features which do not qualify as malformations, such as absent nasal bone or soft markers, especially when there are multiple of these, might also suggest an aneuploidy [6,7,11]. Lastly, ultrasound growth parameters can be valuable. The growth restriction pattern associated with chromosomal anomalies can be present already in the first trimester. The head-to-trunk ratio for crown–rump length has been proposed as a means of formal evaluation, showing a statistically significant increase in cases detected with a trisomy of chromosomes 18 and 13 and triploidy [24].

In recent years, with the introduction of new technologies, it has been possible to correlate the onset of fetal effusions with genetically determined conditions, such as RASopathies, inborn errors of metabolism, musculoskeletal disorders and others. When an ultrasound detection of hydrops fetalis is performed, it is essential to perform a systematic morphological study of the fetus, to evaluate the possible presence of structural anomalies, which could represent the cause of the effusions, or further features which could be ascribed to a syndromic picture or a sequence.

## 3. Genetic Investigations

Chromosomal and genetic disorders represent the most common cause of fetal hydrops, albeit with different epidemiology patterns across trimester I, II and III of pregnancy [7]. In 2015, due to the prevalence of this ultrasound finding and the importance of defining the etiopathogenesis of effusions, the Society of Maternal–Fetal Medicine defined the investigations to be carried out in the case of the gestation of a fetus with hydrops [2]. They included the evaluation for alloimmune haemolysis, fetal anemia, fetal echocardiography, infectious disease tests, and a set of genetic analyses performed on invasive prenatal samples (chorionic villus, amniotic fluid or fetal blood).

### 3.1. Cytogenetic Analyses

The current first-line genetic assessment for fetal hydrops involves rapid aneuploidy detection for trisomies 13, 18, 21 and sex chromosome anomalies with Quantitative Fluorescent Polymerase Chain Reaction (QF-PCR), followed by standard karyotype and chromosomal microarray analysis (CMA) [6,7]. Fetal hydrops occurring in the first trimester can be the sole observable alteration in fetuses with polyploidy. In such cases, non-invasive prenatal testing may yield low risk results, due to limitations of the technology used.

The rate of chromosomal anomalies is higher in the first trimester, when it accounts for up to 70% of the cases of hydrops, and progressively decreases in cases with trimester II (20%) or trimester III (5%) onset [7]. These differences may be due to the early effect on embryo fetal development in most chromosomal anomalies, the non-viability of most affected fetuses presenting early hydrops and to the rates of pregnancy termination in cases of trimester I fetal hydrops and in pregnancies with chromosomal anomalies. It has been reported that fetuses with NIHF in the absence of chromosomal abnormalities have a more favorable outcome [25]. However, even after excluding hydrops caused by chromosomal anomalies and genomic syndromes, the majority of the cases remain without causative diagnosis [11,26]. Notably, the incremental diagnostic yield of chromosomal microarray analysis, compared to karyotyping, amounts to 4.9% (range 3.68–6.13%) in fetuses with isolated NIHF [27]. The prognostic role of pathogenic submicroscopic chromosomal imbalances on the viability of the fetus is less defined, but most are associated with a high risk of developmental disorders and structural anomalies [27].

In the original Society of Maternal–Fetal Medicine document, less specificity was provided regarding the possibility of carrying out in-depth studies of molecular genetics, including the search for sequence variants in specific groups of genes [2].

### 3.2. Molecular Investigations: Customized Panels

In recent years, due to the widespread availability of prenatal molecular testing approaches, cases that did not present cytogenetic anomalies have been increasingly, but variably, subjected to further investigations. Due to the lack of definite guidelines, the access to further testing depends on the discretion of the referring clinician, and on socioeconomic factors involving individuals, families and local regulations of the healthcare system.

Molecular testing for RASopathies has been extensively performed in fetuses presenting with hydrops. RASopathies are a group of conditions who share a dysregulation in Ras-/mitogen-activated protein kinase (RAS-MAPK) signaling, frequently presenting with clinical overlap. The associated conditions can have autosomal dominant or recessive modes of inheritance. For dominantly inherited conditions, the causative variants may arise de novo or be inherited. In recent years, the application in research of genome-wide sequencing approaches has allowed the identification of an increasing number of genes linked to these conditions, progressively included in targeted panels in diagnostics [28]. RASopathy prenatal findings are represented by lymphatic dysplasia—possibly presenting as increased nuchal translucency, cystic hygroma, hydrops fetalis or effusions—along with cardiovascular malformations and polyhydramnios. Depending on the selected cohort and ultrasonographic findings, the incremental diagnostic yield of RASopathy panels—after excluding chromosomal anomalies by karyotyping and/or chromosomal microarray—can vary between 7% and 41% (35% in fetal hydrops, with a *p* value of 0.0001) [29], suggesting that it should be offered, especially in fetuses detected with lymphatic anomalies. For this reason, the next-generation sequencing RASopathy panel is the most frequently offered molecular test in fetuses with effusions. In particular, in the largest reported cohort of fetuses detected with RASopathies, 42% showed hydrops (43% of them presenting with a pathogenic variant in *PTPN11*, while an additional 39% harbored pathogenic variants in *RIT1*, *HRAS*, *RAF1* and *LZTR1*). The onset and prevalence of the prenatal phenotype was varied. Among RASopathy-panel-positive fetuses with hydrops, in 10%, it was detected in the first trimester, and in 90%, the hydrops was still present in the 2nd and 3rd trimester [29].

While RASopathies represent the most commonly investigated monogenic disorders in fetal hydrops, associations with inborn errors of metabolism, musculoskeletal disorders, lymphatic, neurodevelopmental, hematologic diseases and cardiovascular malformations are reported in the scientific literature [30]. The investigation of these additional conditions is less standardized, with marked inhomogeneity in the inclusion in the diagnostic process for fetal hydrops. 

### 3.3. Molecular Investigations: Exome Sequencing

Exome sequencing is a useful tool for non-specific clinical phenotypes and allows the identification of causative single-nucleotide variants in several cases, and is increasingly being offered in the prenatal setting. The diagnostic yield of prenatal exome sequencing in fetuses with ultrasound anomalies, with previous negative cytogenetic testing, amounts to about 19% (range 9–47%) [31].

In particular, promising studies have been reported regarding the diagnostic yield of exome sequencing in the fetuses detected with fluid collections, especially in the case of fetal hydrops [29,32,33,34,35,36], without apparent correlation between the diagnostic yield and the severity of effusions [33]. A recent meta-analysis performed by our group highlighted that the diagnostic yield in this set of fetuses was particularly high compared to cases with other isolated ultrasound anomalies, reaching 30.81% (range 28.56–33.07%) [27]. As expected, the molecular diagnoses are highly heterogeneous, and include, even in apparently isolated cases, RASopathies, other lymphatic dysplasias, musculoskeletal disorders and inborn errors of metabolism. Among these, biallelic variants in the *PIEZO1* gene (MIM*611184), associated with a form of lymphatic dysplasia (Lymphatic Malformation 6, MIM#616843), appear to represent the most frequent monogenic cause of NIHF [37,38]. Collectively, current evidence suggests that the systematic investigation of all monogenic causes of hydrops might be beneficial, even in apparently isolated cases with no anamnestic risk factors.

## 4. Prognosis, Management and Treatment

The identification of the underlying cause is possible in 60–85% of cases, including postnatal evaluation [39], and is pivotal in guiding subsequent clinical management. Current treatment options can prevent further damage to a certain extent, but can rarely interrupt the pathophysiological process or resolve previous lesions. Three groups can be identified based on etiology and treatment options [2,6]: (1) cases in which fetal therapy is available; (2) cases with nonviable conditions (lethal prognosis) and (3) cases in which survival is possible, albeit with uncertain or likely poor prognosis. This category includes cases with an undetermined etiology [2,6].

In cases with treatment options, immediate referral to dedicated fetal medicine institutions is recommended. For immune fetal hydrops, fetal transfusions per transabdominal umbilical vein puncture can reduce the progression of the condition. If fetal tachyarrhythmias (either primitive or resulting from a different condition) are present, depending on the underlying cause, the first-line transplacental therapy throughout the maternal administration of drugs can be proposed, with intra-amniotic administration as a second-tier approach in non-responders [40]. Treatment options differ for fetal supraventricular and ventricular tachyarrhytmias [41]. In particular, in supraventricular tachyarrhythmias (including the more common atrioventricular re-entry tachycardia and atrial flutter), digoxin, sotalol, flecainide and more rarely amiodarione can be suggested, with the aim of slowing the ventricular rate [41]. It is known that, in fetuses with arrhythmias, some variables are associated with an increased risk of developing hydrops, such as early gestational age at the time of the diagnosis, high ventricular rates and incessant tachycardia [41]. These parameters are involved in defining the fetal management, including medical treatment and emergency delivery. In the rare case of fetal ventricular arrthythmias, the maternal injection of magnesium sulfate is usually considered the first-line therapy, but the administration of lidocaine, dexamethasone, beta-blockers, flecainide and amiodarone have also been reported [41].

In cases with cardiac structural anomalies, a limited, but relevant, fraction presenting isolated stenosis/atresia of the outflow tract valves without other structural anomalies (severe aortic stenosis, pulmonary stenosis with intact ventricular septum) can benefit from in utero endovascular cardiac intervention [42]. In cases of ascertained fetal infection with hydrops as one of the clinical signs, tertiary prevention approaches with maternal oral administration of placental-crossing antimicrobial drugs have been proposed, and in some cases, demonstrated to reduce the progression of the fluid imbalance [43]. For some genetic conditions, especially lysosomal storage disorders, in utero targeted treatments are available [44]. These can include trans-umbilical infusion of the lacking enzyme, such as in Pompe disease [45], or attempts at gene therapy with various administration routes (intravenous, intracisternal, intramuscular). Recently, it has been demonstrated that MEK inhibitors reduce the lymphatic involvement described in individuals diagnosed with RASopathy, but only postnatal reports are available [46].

For cases with nonviable conditions and lethal prognosis, aside from pregnancy termination, only comfort care can be offered [2].

The management of the remaining cases, for which the prognosis is uncertain, is more challenging. Especially in cases of undetermined etiology, counseling sessions should focus on the likelihood of adverse pregnancy outcomes [2,6]. Needle drainage of fetal effusions can be performed, with variable results. This approach can be useful to reduce the compression effect exerted by large effusions (usually pleural) and possibly prevent pulmonary hypoplasia or heart failure, but might not be resolutive. Compared to the original classification based on fetal viability and treatment options [2], the increasing application of molecular investigations in diagnostic settings, and the genotype–phenotype correlations emerging from research, studies have yielded precious information to reduce the uncertainties in the class of pregnancy without lethal prognosis, but with no specific treatment available. As opposed to past decades in which only large chromosomal anomalies with an unfavorable prognosis could be investigated, the identification of a monogenic condition might have specific and unexpected prognostic consequences. For example, among apparently isolated prenatal monogenic lymphatic dysplasias, some, such as Hennekam syndrome (MIM #235510), due to biallelic pathogenic variants in *CCBE1* (MIM *612753), can be associated with severe outcomes and systemic postnatal involvement (including intellectual disability) [47], while the apparently common form of lymphatic developmental disorder associated with biallelic pathogenic *PIEZO1* variants often displays a favorable outcome after birth, with the possible resolution of the effusions and edema, if the fetal picture has not resulted in critical sequelae in utero [37,38].

## 5. Discussion

The application of second-generation sequencing technologies has revolutionized many approaches in clinical genetics, allowing the rapid search for causative variants in a large number of genes with a single examination. The short turnaround time has also made its application possible in the prenatal setting. The incremental diagnostic rate of prenatal exome sequencing compared to standard and molecular cytogenetic testing in fetuses with ultrasound anomalies is about 19% [31]; this percentage is comparable to the detection rate of chromosomal microarray analyses, which is why it may be appropriate to perform sequencing analyses in selected cases with structural anomalies and non-conclusive karyotype and chromosomal microarray analyses.

More specifically, the incremental diagnostic yield of exome sequencing amounts to 30.81% in cases of fetal hydrops, showing a consistent increase compared to other ultrasonographic findings [27]. In a more recent paper, Norton and colleagues compared the detection yield of the NGS RASopathies panel, inborn errors of the metabolism panel, the NIHF panel and exome sequencing, highlighting a consistently increased yield in the latter test, with a rate of 29% [35], and a high percentage of cases with variants of potential clinical significance had been highlighted in further series [30]. However, an important limitation was represented by the heterogeneity of the selected samples, which did not exclusively include fetuses with hydrops, but also cases with a single effusion, increased nuchal translucency, cystic hygroma or in which further structural anomalies were present. Although it is useful to avoid the detection of variants in candidate genes or variants of uncertain clinical significance due to interpretative difficulties and the psychological impact that these findings can have on the couple, targeted gene panel sequencing should not be considered as the most appropriate test in conditions in which the phenotype is nonspecific, as in these cases. A summary of the diagnostic yield of different genetic testing approaches in cases of fetal hydrops is provided in Table 1.

The assessments that can be performed in the prenatal setting are always partial, since it is not possible to evaluate in detail the presence of dysmorphisms or predict developmental delay in the absence of central nervous system anomalies. For this reason, genomic investigations are desirable and allow us to obtain a better diagnostic yield, which is essential to allow the couple and clinicians to manage the pregnancy in the most efficient way.

In general, investigating the personal and family history of the pregnant woman always plays a critical role. Many monogenic disorders follow an autosomal recessive trait of inheritance, including some forms of lymphatic dysplasia and metabolic disorders. An anamnesis of parental consanguineity can be decisive in defining the molecular diagnostic algorithm, with the use of a single-nucleotide polymorphism array instead of a comparative genomic hybridization array preferred, and with this making the a priori risk of obtaining a positive result from the exome sequencing higher.

Considering that the incremental diagnostic yield of CMA compared to karyotyping is 4 times lower than the incremental diagnostic yield of exome compared to CMA, it would be appropriate to consider the possibility of performing CMA and exome sequencing in parallel, in order to reach an earlier diagnosis.

One of the main pitfalls of performing exome sequencing in the prenatal setting occurs due to limitations in the genotype–phenotype correlation of non-conclusive results, with this producing a considerable amount of data of non-univocal interpretation. In particular, according to the literature, variants of unknown significance can be identified in 10.47% (confidence interval 9.14–11.79) of fetuses detected with non-immune fetal hydrops [27]. It is known that parental analysis is pivotal for the interpretation of the obtained results; therefore, during pre-test genetic counseling, this topic has to be extensively discussed with the couple before performing the molecular analysis. If it is not possible to access parental blood, the decision to offer exome sequencing should be carefully explored. 

Anyway, genetic diagnostic workflows for fetuses with effusions should consider diagnostic rate, turnaround time, management of variants of unknown significance, economic factors and public health concerns. The few reported series suggest that in cytogenetic-negative fetuses detected with body fluid anomalies, the information provided by exome sequencing can guide genetic counseling, defining the causes of the ultrasound picture and possible postnatal features. These notions are useful for predicting the prognosis and organizing the most appropriate management of both pregnancy and the neonatal period.

In the past, genetic testing was mainly limited to the identification of large chromosomal anomalies, which would usually result in a poor prognosis. More recently, a shift in the implications of the possible result can be noted. The increasing amount of data on monogenic conditions and the availability of prenatal exome sequencing have considerably expanded the array of possible results from genetic testing, with varied and specific implications. Along with an increase in the diagnosis of non-viable forms (e.g., fetal hydrops due to a severe form of skeletal dysplasia), molecular testing can now identify a significant number of conditions with possible treatments (either approved or under investigation). Lastly, genetic results can guide counseling and management in cases with non-lethal genetic conditions for which no specific treatment is available. While this field is characterized by marked uncertainty, the growing number of viable monogenic lymphatic dysplasias with postnatal regression shows how, even in the absence of a targeted therapy, a genetic diagnosis might suggest a more favorable outcome, providing crucial information for fetal medicine specialists and counselors, and guiding prospective parents towards a properly informed choice.

## 6. Conclusions and Future Directions

Currently, after the detection of fetal fluid collections (hydrops, hygroma, hydrothorax, ascites) a prenatal invasive procedure is proposed to the couple. After negative cytogenetic analyses, 80% of fetuses remain without diagnosis. Although there is no clear indication to perform next-generation sequencing panels, some clinicians recommend further investigations when cytogenetic tests are inconclusive. Compared to panels of specific gene groups, exome sequencing provides a higher incremental diagnostic yield for non-immune hydrops fetalis and should be routinely considered in fetuses with effusions, since it allows for the obtaining of a diagnosis in 30% of fetuses with a normal karyotype and negative CMA. Genetic testing is now crucial in identifying not only cases with an unfavorable prognosis, but also cases presenting possibly favorable outcomes with in utero interventions or even targeted therapies.

## Figures and Tables

**Table 1:** The diagnostic yield of genetic testing, based on the trimester of onset of the phenotype.

Genetic Analysis	Incremental Diagnostic Yield for Trimester of Diagnosis		
	I Trim	II Trim	III Trim
Karyotype	70% [7]	20% [7]	5% [7]
CMA	5% [27]		
Rasopathy Panel	15% [29]	30–40% [29]	30–40% [29]
Exome Sequencing	5% [7]	20–30% [7,27]	20–30% [7,27]

## Data Availability

No new data were created or analyzed in this study. Data sharing is not applicable to this article.

## Abbreviations

The following abbreviations are used in this manuscript:

CMA	Chromosomal Microarray Analysis
HF	Hydrops Fetalis
NIHF	Non-Immune Hydrops Fetalis
WES	Whole Exome Sequencing
